# Maternal Nutritional Deficiencies and Small-for-Gestational-Age Neonates at Birth of Women Who Have Undergone Bariatric Surgery

**DOI:** 10.1155/2017/4168541

**Published:** 2017-09-10

**Authors:** J. Hazart, D. Le Guennec, M. Accoceberry, D. Lemery, A. Mulliez, N. Farigon, C. Lahaye, M. Miolanne-Debouit, Y. Boirie

**Affiliations:** ^1^CHU Clermont-Ferrand, Service de Nutrition Clinique, CRNH Auvergne, Université Clermont Auvergne, 63000 Clermont-Ferrand, France; ^2^CHU Clermont-Ferrand, Service de Gynécologie-Obstétrique, Université Clermont Auvergne, 63000 Clermont-Ferrand, France; ^3^CHU Clermont-Ferrand, Délégation Recherche Clinique & Innovation, 63000 Clermont-Ferrand, France; ^4^INRA, Unité de Nutrition Humaine (UNH), CRNH Auvergne, Université Clermont Auvergne, 63000 Clermont-Ferrand, France

## Abstract

The aim is to compare the prevalence of maternal deficiencies in micronutrients, the obstetrical and neonatal complications after bariatric surgery according to surgical techniques, the time between surgery and conception, and BMI at the onset of pregnancy. A retrospective cohort study concerned 57 singleton pregnancies between 2011 and 2016 of 48 adult women who have undergone bariatric surgery. Small-for-gestational-age neonates were identified in 36.0% of pregnancies. With supplements intake (periconceptional period: 56.8%, trimester 1 (T1): 77.8%, T2: 96.3%, and T3: 100.0%), nutritional deficiencies involved vitamins A (T1: 36.4%, T2: 21.1%, and T3: 40.0%), D (T1: 33.3%, T2: 26.3%, and T3: 8.3%), C (T1: 66.7%, T2: 41.2%, and T3: 83.3%), B1 (T1: 45.5%, T2: 15.4%, and T3: 20.0%), and B9 (T1: 14.3%, T2: 0%, and T3: 9.1%) and selenium (T1: 77.8%, T2: 22.2%, and T3: 50.0%). There was no significant difference in the prevalence of nutritional deficiencies and complications according to surgery procedures and in the prevalence of pregnancy issues according to BMI at the beginning of the pregnancy and time between surgery and pregnancy. Prevalence of micronutritional deficiencies and small-for-gestational-age neonates is high in pregnant women following bariatric surgery. Specific nutritional programmes should be recommended for these women.

## 1. Introduction

The last ten years have seen a significant increase in the number of treatments involving bariatric surgery, particularly in women of reproductive age. Women of reproductive age are especially affected by obesity (6.0% of the 18–24-year age group, 11.1% of the 25–34-year age group, and 15.5% of the 35–44-year age group in 2012) [1]. The prevalence of moderate to severe obesity among women during the periconceptional period increased by 6.0% in 1998 to reach 9.9% in 2010 [2]. Periconceptional obesity is a well-established risk factor in the short and long term with respect to maternal, fetal, neonatal, and infantile complications such as miscarriage, gestational diabetes, preeclampsia, pregnancy-induced hypertension, induction of labour, caesarean deliveries, congenital malformations, prematurity, perinatal mortality, large-for-gestational-age neonates, transfer of newborns to intensive care units, juvenile obesity, and type 2 diabetes in mothers [3–5]. Multidisciplinary care combined with therapeutic lifestyle changes forms the first-line treatment for obesity [6]. Should this approach fail, defined by unsatisfactory weight loss over time, bariatric surgery is considered the most effective treatment available in terms of weight loss, the improvement or remission of comorbid conditions, and an increase in survival rates and quality of life in the long term [7–11]. This technique is indicated to treat patients presenting with morbid (BMI ≥ 40 kg/m^2^) or severe (BMI ≥ 35 kg/m^2^) obesity associated with at least one comorbid condition that is likely to improve after surgery (high blood pressure, obstructive sleep apnoea hypopnea syndrome, and other severe respiratory disorders, severe metabolic disorders, particularly type 2 diabetes, disabling bone and joint illnesses, and nonalcoholic steatohepatitis) as a second-line therapy prescribed after the failure of a multidisciplinary care programme carried out effectively over a period of six to 12 months [12]. The recommended surgical techniques include those based exclusively on gastric-restriction techniques: adjustable gastric band (AGB), sleeve gastrectomy (SG), and those that are combined with gastrointestinal malabsorption: gastric bypass (GBP) and biliopancreatic diversion surgery (BPD). Between 2005 and 2014, the number of bariatric surgery procedures conducted in France quadrupled, while AGB surgery has seen a significant decline in favour of SG, which is now the predominant technique followed by GBP and AGB [13, 14]. Of bariatric surgeries performed on 267,466 patients from 2005 until 2014, 86.4% were conducted on women with an average age of 40.3 years [13]. The growth of bariatric surgery among women of reproductive age is associated with an increase in the number of women becoming pregnant after this surgical procedure, raising issues for clinicians concerning the impact of bariatric surgery on pregnancy progress and screening procedures. Bariatric surgery lowers the risk of preeclampsia and gestational diabetes but increases the risk of maternal anaemia. Concerning neonatal complications, it could lower the risk of caesarean deliveries and large-for-gestational-age neonates but increase the risk of premature deliveries, small-for-gestational-age neonates (SGA), admission to neonatal intensive care units, and neonatal death [15–18]. Obesity is a risk factor for nutritional deficiencies that may be increased by both bariatric surgery and pregnancy-induced physiological changes [19, 20]. To our knowledge, no French study is reporting nutritional assessments in women after bariatric surgery during pregnancy. 

The purpose of this study was to describe and compare the prevalence of maternal micronutrient deficiencies and obstetric and neonatal complications in pregnancy after bariatric surgery in terms of surgical technique, the time between surgery and conception, and BMI at the onset of pregnancy.

## 2. Methods

### 2.1. Background

Nutritional care of patients whose pregnancy was monitored at the Clermont-Ferrand Centre Hospitalier Universitaire (CHU) took the form of medical checkups in the CHU's Clinical Nutrition and Obstetrics Depts. The Clinical Nutrition Dept. was responsible for preoperative and postoperative assessments. Periconceptional and prenatal assessments were carried out jointly by the Clinical Nutrition and Obstetrics Depts. The* Association des Utilisateurs de Dossiers Informatisés en Pédiatrie, Obstetrique et Gynécologie* (AUDIPOG) dossier is a common, electronic perinatal healthcare record intended for maternity units that collect all the data required for activities by healthcare professionals and patient monitoring systems. The individual, anonymised datasets were collected from two information sources: printed patient records at the Clinical Nutrition Dept. and the computer backup of AUDIPOG records at the Obstetrics Dept. (keywords: “sleeve gastrectomy”, “by-pass”, and “gastric band”).

#### 2.1.1. Design and Eligibility Criteria

A retrospective cohort study was carried out on adult women with a history of bariatric surgery who became pregnant between July 2011 and March 2016 and who were monitored in the Clinical Nutrition or Gynaecology-Obstetrics Depts. at Clermont-Ferrand University Hospital (CHU). Pregnancies that occurred after the removal of an AGB and those that were not monitored at the Clermont-Ferrand CHU were excluded.

#### 2.1.2. Data Collected by the Researchers

*Sociodemographic data* included date of birth and professional activity.

*Anthropometric data *included height (metres) and weight (kg) before the operation, at the onset of pregnancy, and at each trimester of pregnancy. Body weight was measured at each checkup to the nearest 0.1 kg, with patients in their underwear and with their shoes off and the local scales calibrated. The patient's height and weight at the time of bariatric surgery were used to calculate their preoperative BMI (kg/m^2^). Weight gain during pregnancy was defined as the weight on delivery less the weight at the onset of pregnancy.

*Medical histories *were collected at the time of the bariatric surgery. Anamnestic data included medical, obstetric, and surgical histories together with information on medical treatments. Data on the presence of cardiovascular risk factors such as high blood pressure (blood pressure greater than or equal to 140 and/or 90 mmHg, checked at least twice, WHO definition), type 2 diabetes (fasting blood glucose level greater than or equal to 1.26 g/L, taken twice), and age at the onset of weight gain were collected from the medical histories. Obstetric history included the number and outcome of pregnancies (miscarriages, therapeutic abortion, and births) and parity.

*Surgical data* included the date and type of surgical procedure. The types of surgical procedure included restrictive (AGB and SG) and mixed (GBP) techniques. The date of the operation was used to calculate the time that elapsed between surgery and conception. Data on surgical complications, compliance with nutritional supplementation, minimum postsurgery weight and the time taken to reach this weight, and monitoring frequency (number of medical consultations with nutritionist doctor per year) were collected during patient follow-up.

*Nutritional status *was determined using nutritional supplements, recording the start and finish dates of prescriptions and compliance with supplement intake. Fasting levels of haematological, biochemical, and micronutritional parameters in venous blood were recorded before and after surgery, during the periconceptional period, and at each trimester of pregnancy. Standards specific to pregnant women were used to interpret biological parameter values (Table 1) [21]. Women's standards were used in the absence of any standard specifically designed for pregnant women: vitamins B1 and B6 during the three trimesters of pregnancy [first trimester (T1), second trimester (T2), third trimester (T3)] and vitamin C in T1 and T2.

Blood samples for nutritional dosages were analyzed in the laboratory of the Clermont-Ferrand CHU. Concerning nutritional supplements, systematic supplements contain vitamins (A, B1, B2, B6, B12, C, D3, E, B5, B8, B9, and PP), calcium, magnesium, phosphorus, iron, manganese, copper, and zinc and specific supplements are prescribed in case of deficiency.

Patient* pregnancy-related parameters *were collected at each trimester of pregnancy and included maternal weight gain, gestational age (based on the craniocaudal length recorded on the fetal ultrasound), blood pressure, proteinuria, and stage of pregnancy. Ultrasound data included craniocaudal length, biparietal diameter, abdominal circumference, estimated fetal weight, the growth percentile estimated using Hadlock's formula, and the estimated abdominal perimeter percentile. Anomalies were interpreted based on AUDIPOG criteria. All data on comorbid conditions or complications that appeared during pregnancy and postpartum were collected. The pregnancy-induced hypertension diagnosis was defined by a systolic blood pressure greater than or equal to 140 mmHg and/or diastolic blood pressure greater than or equal to 90 mmHg, isolated, and appearing after 20 weeks of pregnancy based on the recommendations of the International Society for the Study of Hypertension in Pregnancy. Preeclampsia was defined by the presence of chronic or pregnancy-related hypertension and proteinuria greater than or equal to 300 mg per day. Gestational diabetes was defined by the presence of two pathological values identified after oral hyperglycemia detected by an O'Sullivan screening test or by a blood glucose level greater than or equal to 2 g/L during the O'Sullivan test. An intrauterine growth restriction (below the 10th percentile) and its severe form (below the 3rd percentile) were recorded. The outcome of the pregnancy was recorded (miscarriage, birth, and medical termination of pregnancy). According to the AUDIPOG formula, hypotrophy was defined by a small weight for gestational age (SGA) below the 10th percentile and severe hypotrophy by a SGA below the 3rd percentile. Concerning birth and delivery, data on the manner of onset of labour (spontaneous or induced by prostaglandins and/or oxytocics), the methods of delivery (vaginal delivery, forceps delivery, and caesarean), and maternal complications such as postpartum bleeding (blood loss greater than or equal to 500 mL within 24 hours of delivery) were recorded. Lifestyle was assessed based on alcohol and tobacco consumption at the onset of pregnancy.

*Neonatal data* included sex, weight and size at birth, blood pH, the Apgar score at 5 minutes, neonatal complications such as shoulder difficulty or dystocia, the use of special procedures such as admission to a neonatal or intensive care unit, and gestational age expressed as weeks of amenorrhea (WA). Gestational age at birth was used to define the level of prematurity (below 37 WA), including severe prematurity (below 32 WA) and extreme prematurity (below 28 WA). Birth weight and AUDIPOG data were used to distinguish between infants of normal weight (between the 10th and 90th percentile), small-for-gestational-age infants (below the 10th percentile), severe small-for-gestational-age infants (below the 3rd percentile), and large-for-gestational-age infants (above the 90th percentile). Apgar scores of less than 7 to 5 minutes of life and arterial blood pH below 7.20 were considered pathological. Therapeutic abortions (TA) administered due to fetal malformations and in utero fetal deaths were also recorded.

### 2.2. Statistical Analyses

In the descriptive analysis, qualitative variables were described by their numbers and percentages while quantitative variables were described by their means, standard deviations, and minimum and maximum values. The Chi-Squared Independence Test was used to compare ratios when theoretical numbers were greater than 5. In cases where this condition was not checked, Fisher's exact test was used. Comparisons of quantitative variable means were performed using the Student's test (2 groups) or by variance analysis based on ANOVA tests (more than 2 groups). All tests were bilateral and the acceptable risk of error was set at 5%. The statistical analyses were performed using Stata 12.0 software (STATACORP, Texas, USA).

## 3. Results

**Figure 1:**
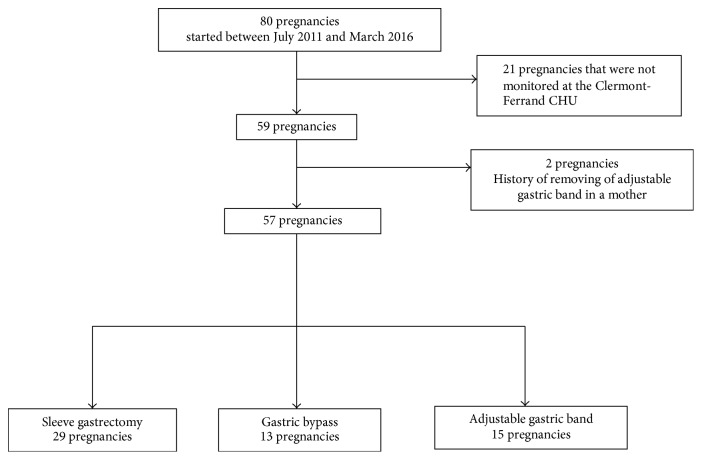
Flow chart.

This study covered all adult obese women who had undergone bariatric surgery and become pregnant between July 2011 and March 2016 and who had been monitored in the Clinical Nutrition and Obstetrics Depts. at Clermont-Ferrand CHU. Out of the 80 pregnancies involving patients with a history of bariatric surgery that began between March 2011 and July 2016, 59 were monitored in the Clinical Nutrition and Obstetrics Depts. at Clermont-Ferrand CHU. Two pregnancies in which the mother had had the AGB removed prior to becoming pregnant were excluded (Figure 1). In all, this study covered 57 singleton pregnancies in 48 patients. A total of nine women had two pregnancies within the study period. Tables 2 and 3 illustrate the maternal, neonatal, and pregnancy-related characteristics.

### 3.1. Maternal Characteristics

A total of 48 patients underwent bariatric surgery with an average preoperative BMI of 47.0 ± 6.0 kg/m^2^. There were a total of 57 singleton pregnancies in these 48 patients who had an average age of 31.0 ± 5.8 years and an average BMI of 30.5 ± 7.4 kg/m^2^ at the onset of pregnancy. A maternal history of AGB, SG, and GBP was recorded in 26.0%, 51.0%, and 23.0% of pregnancies, respectively (Table 2). Postoperative follow-up was conducted on a regular basis in 37.0% of patients, while 23.5% of patients declared having complied with their supplementation plan during the postoperative period. Maximum weight loss between surgery and pregnancy was generally around 52.0 ± 15 kg.

### 3.2. Pregnancy-Related Characteristics

As regards nutritional status, 56.8% of pregnancies involved supplementation during the periconceptional period. Average weight gain during pregnancy was 11.5 ± 5.5 kg. Gestational diabetes and pregnancy-induced hypertension were reported in 18.0% and 4.0% of pregnancies, respectively. Concerning pregnancy outcome, 8.8% (i.e., 5) of outcomes involving three patients resulted in a TA due to malformation, including one case of spina bifida.

### 3.3. Neonatal Characteristics

Five cases of malformations with TA were counted in our study. Three out of five TA involved chromosomal rearrangements (trisomy 21) in the same patient. There was one case of polymalformative syndrome without detection of chromosomal rearrangements. There was one case of spina bifida in a patient who had undergone SG and who presented with a folate deficiency.

No cases of malformation or neonatal death were reported among the 52 neonates. Concerning neonatal complications, 28.0% of newborns were premature, 36.0% were small-for-gestational-age, 4.0% were severely small-for-gestational-age, and 4.0% were large-for-gestational-age.

Concerning nutritional status, 77.8% of pregnancies involved supplementation during the periconceptional period in T1, 96.3% in T2, and 100.0% in T3 (Table 4). The principal micronutrient deficiencies identified were vitamin A (T1: 36.4%, T2: 21.1%, and T3: 40.0%), D (T1: 33.3%, T2: 26.3%, and T3: 8.3%), C (T1: 66.7%, T2: 41.2%, and T3: 83.3%), B1 (T1: 45.5%, T2: 15.4%, and T3: 20.0%), B9 (T1: 14.3%, T2: 0%, and T3: 9.1%), and selenium (T1: 77.8%, T2: 22.2%, and T3: 50.0%). The mean of nutritional values showed no significant difference according trimesters of pregnancy. The rate of haemoglobin decreased significantly between T1 and the other trimesters of pregnancy, which is a physiological evolution due to expansion of plasma volume.

### 3.4. According to Surgical Technique

A few maternal, nutritional, and pregnancy-related parameters revealed significant differences depending on the surgical technique used (Table 5). Women who had undergone a SG had a higher BMI at the time of the surgery (49.0 ± 6.3 kg/m^2^ versus 43.8 ± 4.1 kg/m^2^ for AGB and 43.8 ± 4.4 kg/m^2^ for GBP; *p* = 0.01) and a greater postoperative weight loss (57.6 ± 14.6 kg versus 42.6 ± 16.3 kg for AGB and 49.2 ± 9.4 kg for GBP; *p* = 0.03). The interval between surgery and conception and BMI at the onset of pregnancy were greater in patients with a history of AGB. In T1, there were fewer patients with an AGB who took supplements (AGB: 25.0%, SG: 77.3%, and GBP: 100.0%; *p* = 0.01). The prevalence of neonatal complications and nutritional deficiencies showed no significant difference according to surgical technique.

### 3.5. According to the Time between Surgery and Conception

Among the women who became pregnant within 18 months of bariatric surgery, 47.1% were losing weight versus 7.7% for patients who waited for a period greater than or equal to 18 months (*p* = 0.008; Table 6). Other maternal characteristics and neonatal and pregnancy issues showed no significant difference according to the time between surgery and conception.

### 3.6. According to BMI at the Onset of Pregnancy

The time between bariatric surgery and minimum weight reached was shorter for obese women at the onset of pregnancy (13.4 ± 7.2 months versus 21.6 ± 9.4 months for nonobese patients at the onset of pregnancy; *p* = 0.02). A greater number of obese women were losing weight at the onset of pregnancy (37.0% versus 13.0% of nonobese patients; *p* = 0.03, Table 7). Other maternal characteristics and neonatal and pregnancy issues showed no significant difference according to the maternal BMI at the onset of pregnancy.

## 4. Discussion

Our study demonstrates that women who were pregnant after bariatric surgery have a great risk of presenting with micronutritional deficiencies in vitamins A, B1, B9, B12, and D and selenium in each trimester of pregnancy. Maternal deficiencies in vitamins A, D, B1, B9, and B12 were also identified in the scientific literature [22, 23]. No study was found covering nutritional statuses for vitamin C, selenium, zinc, and magnesium in pregnant women with a history of bariatric surgery.

In accordance with the data in the scientific literature, our study did not reveal any significant association between micronutrient deficiencies and fetal malformations [22]. The prevalence of fetal malformations in our study was higher than in the literature, which can be explained by the fact that three out of five TA involved chromosomal rearrangements in the same patient [24]. One pregnancy was terminated following an antenatal diagnosis of spina bifida in a patient who had undergone SG and who presented with a folate deficiency. Pelizzo et al. described four cases of neural tube anomalies in patients deficient in folates, three of whom had a history of GBP and one had a history of BPD [25–27]. Given that neural tube anomalies are more frequent in overweight and obese women than in normal-weight women, it is impossible to deduce whether these malformations were caused by the folate deficiency [28].

We have demonstrated that all the surgical techniques investigated in the study, including restrictive procedures, showed a strong relationship with micronutritional deficiencies, with these findings supporting those found in the scientific literature [23]. It should be noted, however, that the prevalence of treatments using nutritional supplements in the first trimester of pregnancy in our study differed significantly depending on the techniques used. All the women with a history of GBP were actually taking supplements based on the recommendations on good medical practice (professional agreement). French and international recommendations state that, in the event of pregnancy, and in particular after gastric bypass surgery, it is advisable for women to take supplements of iron, calcium, folates, vitamin B12, and vitamin D. In accordance with international recommendations, treatment with folate supplements should be set up as soon as a woman expresses the desire to become pregnant [12]. It is also advised that the multidisciplinary healthcare team should programme a nutritional monitoring plan during the pregnancy and postpartum period (professional agreement). The prevalence of the observed deficiencies illustrates the need for regular medical checkups after bariatric surgery coupled with a dietetic and nutritional, clinical, and biologic assessment during the periconceptional period or, failing this, at the onset of pregnancy [12]. In accordance with our findings, several studies have reported that approximately fifty percent of patients fail to take their vitamin supplements in the long term, a fact that could impact on the health of a pregnancy [29]. Patient compliance and ongoing information and support provided by caregivers are, therefore, important components of the efforts set up to prevent complications and deficiencies.

Regarding neonatal complications, the high prevalence of small-for-gestational-age neonates identified in our study mirrored the findings of the study by Johansson et al. that highlighted an increased risk of small-for-gestational-age neonates (defined by a birth weight below the 10th percentile) in patients with a history of bariatric surgery [17]. In the meta-analysis performed by Galazis et al., the incidence of small-for-gestational-age neonates in patients who had undergone bariatric surgery was higher with a level of risk that was approx. 80% greater than that for obese pregnant women (OR: 1.93 [1.52–2.44]; *p* < 0.001) [15]. These findings have been confirmed by other studies [30–34]. In our study, the prevalence of SGA and prematurity is greater than previous published data which could be explained by the heterogeneous definitions of these terms. Indeed, in the meta-analysis of Galazis et al., only seven studies out of eleven defined* small neonates* as having an SGA below the 10th percentile, similar to that reported in our study. In eight studies, prematurity was defined as birth before the 37th week of gestation, but in four studies no definition was provided. Moreover, in some previous published data, we do not know the nutritional status of these women at the beginning and during the pregnancy on one hand and their weight dynamics between the surgery and the conception on the other hand [15]. In clinical practice, a history of bariatric surgery should therefore be considered a risk factor for SGA. Maternal undernutrition and micronutrient deficiencies resulting from caloric restriction as part of bariatric surgery could be one of the mechanisms underlying SGA [35]. While nutritional status was not assessed, Johansson et al. suggested an association between the findings and pregnancy-related malnutrition concerning, in particular, deficiencies in iron, vitamins B12, and folates [17]. We put forward the hypothesis that special attention should be paid to assessing the protein-energy status in such patients. Protein deficiencies are frequently identified after bariatric surgery, especially after a GBP [36]. In keeping with the findings reported in the scientific literature, our study did not reveal any significant difference in the prevalence of small-for-gestational-age neonates in relation to surgical techniques [15]. Nevertheless, two studies reported that the prevalence of SGA was lower in pregnant women fitted with an AGB compared to obese women who had not undergone surgery [37, 38]. A case-control study reported a higher prevalence of SGA after GBP compared to restrictive surgical weight loss techniques (AGB and SG) [39]. These studies made no mention of preoperative BMI or of the time that elapsed between surgery and conception. This could indicate that the patients did not share similar weight dynamics in terms of the techniques used. Could restrictive surgical weight loss techniques really involve a lower risk of SGA or might it simply be that the weight dynamic of these patients is a decisive factor in assessing the risk of SGA? As the greatest weight loss occurs in the 12- to 18-month period after bariatric surgery, it is recommended that this time period prior to becoming pregnant should be respected so as to minimise the maternal, fetal, and neonatal complications associated with potential nutritional deficiencies and to optimise weight loss [40–43]. In line with the data found in the scientific literature, the prevalence of complications did not differ significantly according to time [44, 45]. This supports the hypothesis that the criteria for stabilisation of patient weight and correction of nutrient deficiencies would be of greater value when deciding on starting a pregnancy than the criteria for the time between surgery and conception. It should also be noted that rapid postoperative weight loss results in the distortion of body image. Weight gain during pregnancy may generate anxiety and decompensation of eating disorders, and restrictive behaviours in particular, due to the fear of regaining weight [41]. Waiting for the weight to stabilise prior to becoming pregnant could help to prevent such complications. The benefits of bariatric surgery in terms of reducing maternal metabolic and cardiovascular risks could be counteracted in the long term by the small-for-gestational-age adverse metabolic and cardiovascular effects [46]. Due to this potential risk of malnutrition and fetal growth restriction, more regular monitoring of fetal growth could be programmed for pregnant women with a history of bariatric surgery. Additional studies will be needed to establish recommendations on clinical-biological monitoring and supplementation treatments for women during both the periconceptional period and pregnancy after bariatric surgery. The prospective multicentric cohort study bAriatric sUrgery Registration in wOmen of Reproductive Age (AURORA) designed to monitor women of reproductive age prior to bariatric surgery and for up to 6 months after pregnancy will make it possible to assess the prevalence and incidence of maternal and neonatal nutritional deficiencies and fetal malformations and will support the establishment of recommendations on good clinical practice for pregnant women with a history of bariatric surgery [47].

Although this study did not include a control group and was of a fairly small cohort size, it is, nevertheless, the largest French study reporting on micronutrient assessments in each trimester of pregnancy. We also took into account the physiological variations in micronutrient rates during each trimester of pregnancy by referring to standardised micronutrient levels that are specific to pregnant women in order to avoid overestimating deficiency prevalence. Even if there is no other data, concerning standards given by Abbassi-Ghanavati et al., some normal intervals are large and population is different from the French pregnant women so that these values are perhaps not completely adequate for French pregnant women.

## 5. Conclusions

Deficiencies in vitamins A, D, C, B1, and B9 and selenium have been identified in women who become pregnant after bariatric surgery. The high prevalence of small-for-gestational-age neonates raises the issue of the protein-energy status of women who fall pregnant after bariatric surgery. These findings underscore the importance of regular monitoring and compliance with long-term nutritional supplement plans after bariatric surgery. These nutritional deficiencies and the high rate of small-for-gestational-age neonates justify the need for systematic screening and the development of specific protocols for pregnant women who have undergone bariatric surgery.

## Figures and Tables

**Table 1:** Biological standards for haematological, biochemical, and micronutritional parameters in pregnant women.

	T1^a^	T2^b^	T3^c^
	min^d^–max^e^		
*Haematology*			
Haemoglobin (g/dL)	11.6–13.9	9.7–14.8	9.5–15
MCV^f^ (fL)	81–96	82–97	81–99
Ferritin (*μ*g/L)	6–130	2–230	0–116
*Biochemistry*			
Albumin (g/L)	31–51	26–45	23–42
*Vitamins *			
A (*μ*mol/L)	1.12–1.64	1.22–1.54	1.01–1.47
B1 (nmol/L)	/^g^	/^g^	/^g^
B6 (nmol/L)	/^g^	/^g^	/^g^
B9 (nmol/L)	5.89–33.99	1.81–54.39	3.17–46.9
B12 (pmol/L)	87.08–323.24	95.94–484.13	73.06–388.2
C (*μ*mol/L)	/^g^	/^g^	51.1–73.81
D (*μ*g/L)	18–27	10–22	10–18
*Minerals*			
Magnesium (mmol/L)	0.66–0.90	0.62–0.90	0.45–0.90
Selenium (*µ*mol/L)	1.47–1.85	0.95–1.85	0.90–1.69
Zinc (*μ*mol/L)	8.72–13.47	7.65–13.47	7.65–11.78

^a^First trimester. ^b^Second trimester. ^c^Third trimester. ^d^Minimum. ^e^Maximum. ^f^Mean Corpuscular Volume. ^g^There are no standards specific to pregnant women.

**Table 2:** Maternal characteristics of 48 patients.

	%^a^ m^b^ ± sd^c^ [min–max^d^]
*Maternal characteristics *(*N* = 48)	
Maternal age (years)	31.0 ± 5.8 [22–44]
Start of weight gain	
(i) Childhood	83.0
(ii) Adolescence	3.0
(iii) Adulthood	14.0
Antecedents	
(i) Type 2 diabetes	3.5
(ii) High blood pressure	15.5
(iii) Active smoking	35.0
Professional activities	
(i) Active or in training	60.5
(ii) Unemployed, at home, or on parental leave	39.5
Bariatric surgery	
(i) Preoperative weight (kg)	129 ± 18.0 [103.0–174.0]
(ii) Preoperative BMI^e^ (kg/m^2^)	47.0 ± 6.0 [38.0–63.0]
(iii) AGB^f^	25.0 (*N* = 12)
(iv) SG^g^	47.9 (*N* = 23)
(v) GBP^h^	27.1 (*N* = 13)
(vi) Postsurgery supplementations	23.5
(vii) Postsurgery monitoring^i^ (per year)	
(i) Regular ≥ 3	37.0
(ii) Irregular [1-2]	24.0
(iii) None, 0	39.0
(viii) Maximum weight loss	52.0 ± 15.0 [30.0–79.0]
Time between surgery and pregnancy (months)	40.7 ± 33.9 [5–130]
Gravidity	
(i) 1	36.8
(ii) 2	17.5
(iii) 3	22.8
(iv) >3	22.9
Parity	
(i) 0	42.1
(ii) 1	29.8
(iii) 2	10.5
(iv) >3	17.6

^a^Percentage. ^b^Mean. ^c^Standard deviation. ^d^Minimum–maximum. ^e^Body Mass Index (kg/m^2^). ^f^Adjustable gastric band. ^g^Sleeve gastrectomy. ^h^Gastric bypass. ^i^Medical consultation with nutritionist doctor.

**Table 3:** Neonatal and pregnancy-related characteristics of 57 pregnancies in 48 patients.

	%^a^ m^b^ ± sd^c^ [min–max^d^]
*Pregnancy-related characteristics* (*N* = 57)	
Nutritional supplementation in the periconceptional period	56.8
BMI^e^ at the onset of pregnancy (kg/m^2^)	30.5 ± 7.4
Maternal weight gain (kg)	11.5 ± 5.5
Pregnancy-induced hypertension	4.0
Gestational diabetes	18.0
Preeclampsia	0
IUGR^f^	3.0
Threat of premature delivery	11.1
Induction of labour	36.1
TA^g^ for malformation	8.8 (*N* = 5)
Postpartum bleeding	3.0
*Neonatal characteristics* (*N* = 52)	
Gestational age at birth (WA^h^)	38.0 ± 2.7 [27.0–41.0]
Birth weight (g)	3026.0 ± 553.0 [1065.0–3900.0]
Prematurity between 32 and 36 WA	11.0
(i) Severe prematurity < 32 WA	3.0
(ii) Extreme prematurity < 28 WA	14.0
AUDIPOG	
(i) Hypotrophy *P* < 10	32.0
(ii) Severe hypotrophy *P* < 3	4.0
(iii) Macrosomia *P* > 90	4.0
Shoulder difficulty	6.0
Shoulder dystocia	3.0
Apgar score < at 7 to 5 min.	12.5
Umbilical blood pH < 7.2	5.0
Transfer to the neonatal unit	12.0
Transfer to the intensive care unit	6.0

^a^Percentage. ^b^Mean. ^c^Standard deviation. ^d^Minimum–maximum. ^e^Body Mass Index. ^f^Intrauterine growth restriction. ^g^Therapeutic abortion. ^h^Weeks of amenorrhea.

**Table 4:** Mean values of parameters and prevalence of maternal nutritional deficiencies during pregnancy (*N* = 57).

	T1^a^	T2^b^	T3^c^	*p* ^d^	*p* ^e^	*p* ^f^
	m^g^ ± sd^h^/%^i^					
Supplementation	77.8	96.3	100.0			
*Haematology*						
Haemoglobin (g/dL)	12.9 ± 1.6/5.0	11.9 ± 1.2/7.1	11.6 ± 1.0/0	0.01	0.001	
Ferritin (ng/mL) *μ*g/L	43.9 ± 42.3/5.9	28.2 ± 34.0/0	35.7 ± 57.8/0			
*Biochemistry*						
Albumin (g/L)	37.8 ± 4.6/13.3	32.5 ± 3.2/5.6	31.1 ± 2.8/0			
*Vitamins *						
A (*μ*mol/L)	1.2 ± 0.19/36.4	1.3 ± 0.25/21.1	1.2 ± 0.5/40.0			
B1 (nmol/L)	125.1 ± 43.8/45.6^j^	138.0 ± 34.5/15.4^j^	134.7 ± 41.8/20.0^j^			
B6 (nmol/L)	85.8 ± 27.0/0^j^	110.6 ± 80.4/0^j^	77.4 ± 20.1/0^j^			
B9 (nmol/L)	21.8 ± 9.8/14.3	22.5 ± 20.0/0	24.0 ± 19.0/9.1			
B12 (pmol/L)	215.0 ± 73.0/0	204.0 ± 78.0/4	184.0 ± 51.0/0			
C (*μ*mol/L)	23.0 ± 18.0/66.7^j^	34.0 ± 21.0/41.2^j^	32.0 ± 19.0/83.3			
D (*μ*g/L)	28.3 ± 18.7/33.3	23.7 ± 18/26.3	30.4 ± 13/8.3			
*Minerals*						
Magnesium (mmol/L)	0.8 ± 0.1/0	0.8 ± 0.1/0	0.7 ± 0.1/0			
Selenium (*µ*mol/L)	0.9 ± 0.2/77.8	0.9 ± 0.2/22.2	0.8 ± 0.1/50.0			
Zinc (*μ*mol/L)	13.1 ± 2.6/0	11.5 ± 1.7/0	10.7 ± 1.4/0			

^a^First trimester. ^b^Second trimester. ^c^Third trimester. ^d^Comparison T1-T2. ^e^Comparison of T1-T3. ^f^Comparison of T2-T3. ^g^Mean. ^h^Standard deviation. ^i^Percentage of deficiency with specific standards for pregnant women. ^j^Percentage of deficiency when no specific standards are used.

**Table 5:** Maternal, nutritional, and pregnancy-related characteristic according to surgical technique.

	AGB^a^ (*N* = 15)	SG^b^ (*N* = 29)	GBP^c^ (*N* = 13)	*p*
	%^d^ m^e^ ± sd^f^			
*Maternal characteristics*				
Weight at the time of surgery (kg)	118.0 ± 11.0	138.0 ± 18.0	118.0 ± 12.0	0.0002
BMI^g^ at the time of surgery (kg/m^2^)	43.8 ± 4.1	49.0 ± 6.3	43.8 ± 4.4	0.01
Maximum weight loss (kg)	42.6 ± 16.3	57.6 ± 14.6	49.2 ± 9.4	0.03
*Pregnancy*				
Time between surgery and pregnancy (months)	64.0 ± 40.0	33.0 ± 28.0	30.0 ± 25.0	0.006
Weight at the onset of pregnancy (kg)	90.0 ± 21.0	87.0 ± 20.0	71.0 ± 12.0	0.02
BMI at the onset of pregnancy (kg/m^2^)	33.5 ± 7.9	31.0 ± 7.8	26.3 ± 4.2	0.03
*Nutritional characteristics*				
Supplementations at T1^h^	25.0	77.3	100.0	0.01

^a^Adjustable gastric band. ^b^Sleeve gastrectomy. ^c^Gastric bypass. ^d^Percentage. ^e^Mean. ^f^Standard deviation. ^g^Body Mass Index. ^h^First trimester. All the maternal, neonatal, nutritional, and pregnancy-related characteristics presented in Tables 2 and 3 were compared according to the surgical technique used. The table only shows results that were statistically significant or were of borderline statistical significance.

**Table 6:** Maternal and pregnancy-related characteristics according to the time between bariatric surgery and pregnancy.

	Period < 18 months	Period ≥ 18 months	*p*
	*N* = 23	*N* = 34	
	%^a^ m^b^ ± sd^c^		
Time between surgery and pregnancy (months)	13 ± 4	57 ± 33.5	<0.0001
Minimum weight after surgery (kg)	81 ± 14	70 ± 9	0.01
Body weight dynamics at the onset of pregnancy			0.008
(i) Weight loss	47.1	7.7	
(ii) Weight stabilisation	53	84	
(iii) Weight gain	0	8.0	

^a^Percentage. ^b^Mean. ^c^Standard deviation. All the maternal, neonatal, nutritional, and pregnancy-related characteristics presented in Tables 2 and 3 were compared according to the time between bariatric surgery and pregnancy. The table only shows results that were statistically significant or were of borderline statistical significance.

**Table 7:** Maternal and pregnancy-related characteristics according to Body Mass Index at the onset of pregnancy.

	BMI^a^ < 30 kg/m^2^	BMI ≥ 30 kg/m^2^	*p*
	(*N* = 27)	(*N* = 30)	
	%^b^ m^c^ ± sd^d^		
Weight at the time of surgery (kg)	122 ± 13	138 ± 20	0.001
Time between surgery and minimum weight (months)	21.6 ± 9.4	13.4 ± 7.2	0.02
Body weight dynamics at the onset of pregnancy			0.03
(i) Weight loss	13	37	
(ii) Weight stabilisation	87	53	
(iii) Weight gain	0	10	

^a^Body Mass Index. ^b^Percentage. ^c^Mean. ^d^Standard deviation. All the maternal, neonatal, nutritional, and pregnancy-related characteristics presented in Tables 2 and 3 were compared according to BMI at the onset of pregnancy. The table only shows results that were statistically significant or were of borderline statistical significance.
